# Study on the multi-wavelength variation of 3C 454.3

**DOI:** 10.1038/s41598-025-93386-7

**Published:** 2025-03-12

**Authors:** Xu Chen, Shaoming Hu, Yunguo Jiang, Jingran Xu, Shifeng Huang, Ruixin Zhou, Hongxing Yin, Difu Guo, Yutong Li, Huaizhen Li

**Affiliations:** 1 Shandong Provincial Key Laboratory of Optical Astronomy and Solar-Terrestrial Environment, School of Space Science and Technology, Institute of Space Sciences, Shandong University, Weihai, 264209 Shandong China; 2 CAS Key laboratory for Research in Galaxies and Cosmology, Department of Astronomy, University of Science and Technology of China, Hefei, 230026 Anhui China; 3 College of Physics and Electronic information, Dezhou University, Dezhou, 253023 Shandong China; 4 Department of Physics, Yuxi Normal University, Yuxi, 653100 Yunnan China

**Keywords:** 3C 454.3, Blazars, Variation mechanism, Color variation, High-energy astrophysics, Computational astrophysics, Time-domain astronomy

## Abstract

The variation mechanism of the blazar is still an open question. In this study, we collect the long-term multi-wavelength data of 3C 454.3 to conduct a comprehensive study. The local cross-correlation functions were computed between the $\gamma$-ray and *R* band fluxes, as well as between *B* and *K* band fluxes. No significant time lags were found among these bands, which suggests that the optical and $\gamma$-ray emissions are co-spatial. The color indices variation behavior showed a redder-when-brighter trend in the lower state, and a saturation state in the higher state. The slope of the linear correlations between the logarithms of synchrotron and inverse-Compton fluxes changed from 0.61 to 3.34 for different band pairs, which could be explained by a model of Doppler-boosted log-parabolic synchrotron emission combined with stable background contamination. The model could also reproduce the spectral energy distributions at different brightness. This study can help us to better understand the variation mechanism of blazars.

## Introduction

Blazars, a subclass within the broader category of active galactic nuclei (AGNs), are characterized by their unique configuration that a relativistic jet is oriented at an extremely small viewing angle relative to Earth-bound observers^46^. Blazars are composed of flat spectrum radio quasars (FSRQs) and BL Lacertae objects (BL Lacs). FSRQs show prominent emission lines compared with BL Lacs^46^. Spectral energy distributions (SED) of blazars have two bumps, one with a lower frequency peak, regarding as the synchrotron component, and another with a higher frequency peak, regarding as the inverse Compton (IC) component, arising from either synchrotron self-Compton (SSC) or external Compton (EC) processes, under the leptonic scenarios (e.g.,^5,6,11,25,40^).

Blazars show rapid and high-amplitude variability in almost all wavelengths ranging from radio to $\gamma$-ray. The variation mechanism continues to pose an intriguing open question. To unravel this, comprehensive multi-wavelength observational campaigns are conducted with the aim of elucidating the intricate emission regions and the complex radiation processes in blazars. Usually, the flux variations observed in blazars can result from intrinsic and extrinsic factors. Intrinsic mechanisms in blazar jets encompass the dynamic behavior of plasma blobs, leading to variable compression, polarization, and emission properties, as well as the generation and propagation of shocks within the jet^22,26,35^. Extrinsic factors predominantly concern the geometric influences resulting from variations in the observer’s line of sight relative to the orientation of the emitting region. These effects, particularly the variable Doppler-boosting of the emitted radiation, significantly influence the observed flux levels, introducing an additional complexity to the interpretation of blazar light curves^12,35,48^.

The FSRQ object 3C 454.3, located at $z=0.859$^16,19^, is a profoundly enigmatic source that exhibits extreme variability across the entire electromagnetic spectrum, ranging from radio frequencies to $\gamma$-rays. In recent years, it has been under intense observation due to its propensity for rapid and vehement outbursts. However, the underlying mechanism driving this dramatic variability remains an unsolved mystery (e.g.,^13,14,21,24,30,38,47,49,53^).

Time lags and correlations between different bands are key to constrain the emission regions and mechanisms.^34^ found that sometimes the optical and $\gamma$-ray flux for 3C 454.3 vary simultaneous, whereas in some other cases the latter seem to be possibly delayed. They also found that the X-ray flux variations lag behind the optical ones. The X-ray lag was also found by^21^. However, the X-ray flux varies simultaneously with the optical during some periods^21,50^, and does not vary with the optical variation during some other periods^3^.

3C 454.3 exhibits a redder-when-brighter (RWB) pattern at a faint state, where its color becomes redder as its brightness increases. However, this trend reaches a saturation point at its most luminous state^14,33,38,54,55^.^2^ explained the color-magnitude variations as highly variable jet emission mixed with slower varying disk emission. The RWB trend is typically understood as a bluer light contamination from the active accretion disk superimposed onto the inherently emissions emanating from the variable jet. In order to explore the mechanisms of color variation, we assembled the multi-wavelength dataset for the object 3C 454.3, spanning from infrared to $\gamma$-ray frequencies, and investigate the correlations among them and the broadband SED. This paper is organized as follows. The data collection and reduction of 3C 454.3 is presented in Section "Observation and data reduction". Sections "Variation"-"Correlations between Synchrotron and inverse-compton fluxes" show the results of the variations, time lag analysis, variability timescale, color variation properties, and variation correlations between synchrotron and IC bands, respectively. An analytical model is proposed and the observation properties are fitted and predicted in Section "The log-parabolic model". Finally, the conclusions are given in Section "Conclusion".

## Observation and data reduction

### Fermi LAT data

**Fig. 1:**
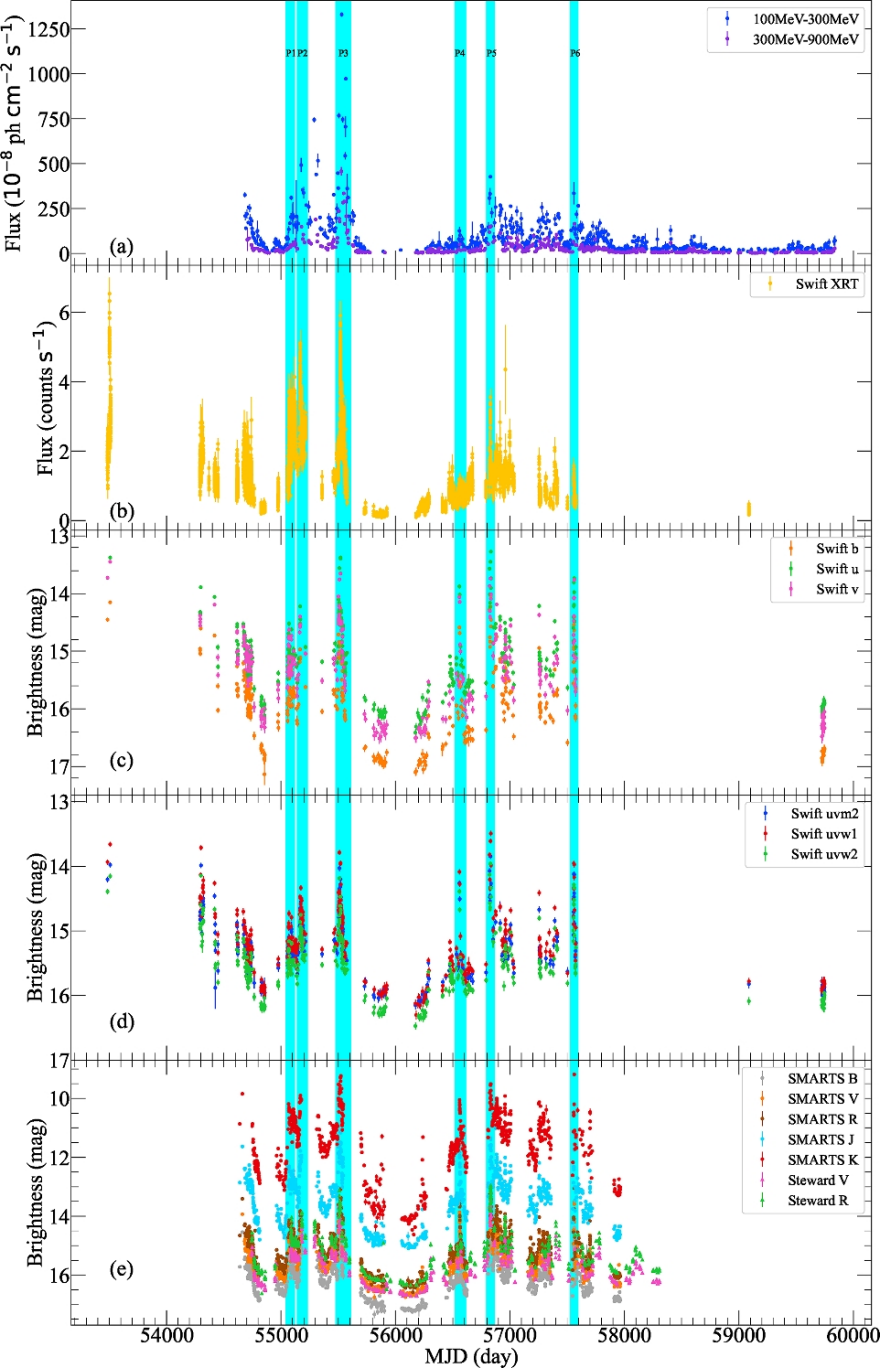
Multi-band light curves of 3C 454.3.

**Fig. 2:**
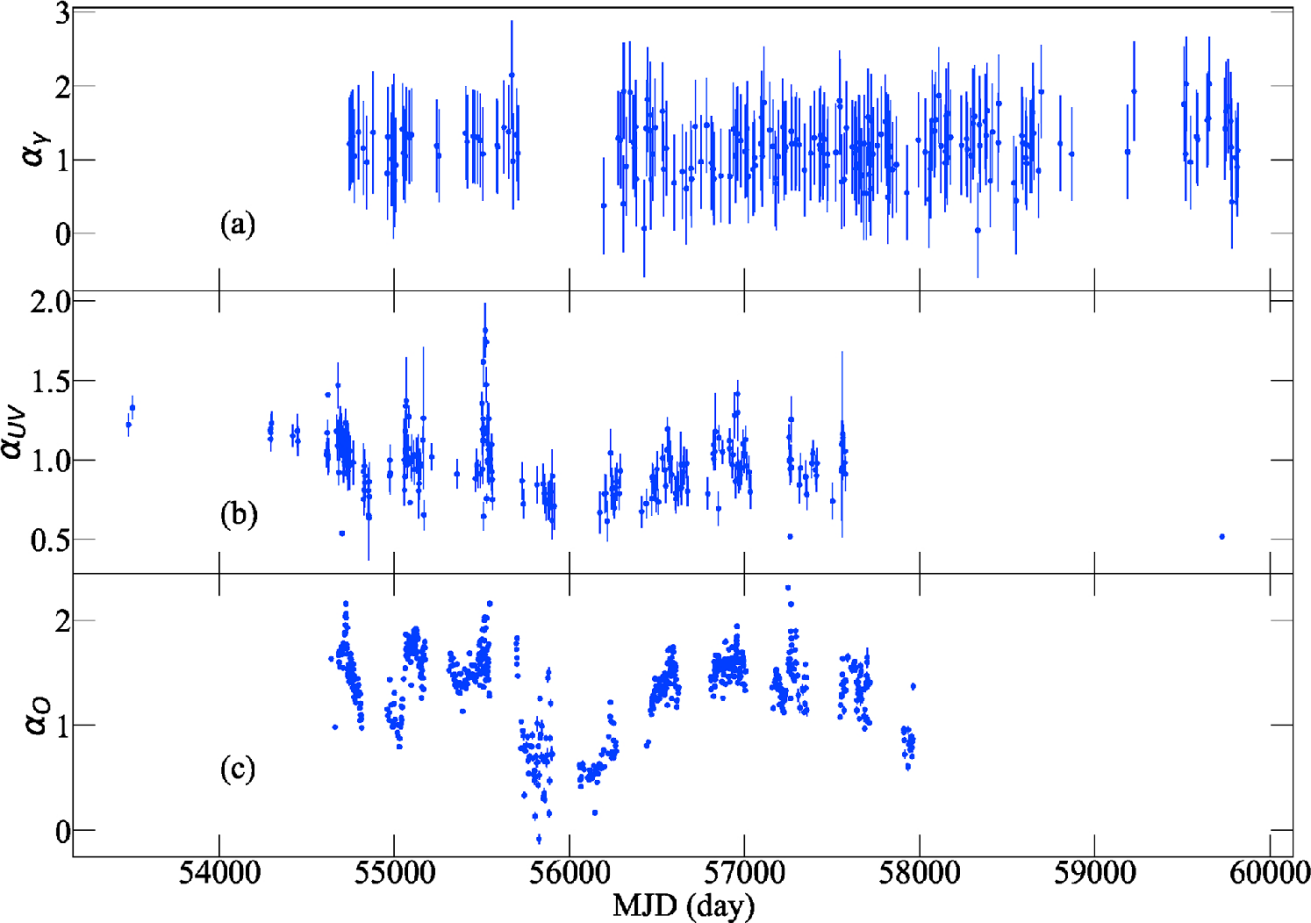
The variation of the spectral index $\alpha _\gamma$, $\alpha _{UV}$ and $\alpha _O$.

All the Pass 8 (P8R3) events data between August 4, 2008 and October 21, 2022 are downloaded from the *Fermi* data server. The events with energy ranging from $100-300$ MeV and $300-900$ MeV are extracted from a region of interest (ROI) of $10^{\circ }$ around 3C 454.3. All the data are processed in the following steps. Events of event class 128 and event type 3 coming from zenith angles smaller than $90^{\circ }$ are selected by the *gtselect* tool. Expression $(DATA\_QUAL>0) \ \& \& \ (LAT\_CONFIG == 1 )$ is used to get good time intervals by the *gtmktime* tool. Tools of *gtltcube* and *gtexpmap* are executed to generate the exposure map. Files $gll\_iem\_v07.fits$ and $iso\_P8R3\_SOURCE\_V3\_v1.txt$ are used by the model editor tool *make*4*FGLxml*.*py* to generate the XML model file, in which spectral parameters were fixed for sources beyond $5^{\circ }$. The diffuse source responses are then computed. An overall fit is performed using the DRMNGB optimizer, and the result is recompute using MINUIT to find more accurate results. After that, data are selected with the TS value greater than 10. The 3-day binned light curves of the integral flux are plotted in panel (a) of Figure 1. The $\gamma$-ray spectral index $\alpha _\gamma$ is defined as $\alpha _\gamma = -\log [F_{300-900 \mathrm MeV}/F_{100-300 \mathrm MeV}]/\log 3$. The time series of the spectral index is plotted in the panel (a) of Figure 2.

### Swift Data

We gathered the X-ray data from the X-Ray Telescope (XRT) and the Ultra Violet and Optical Telescope (UVOT) of Swift. XRT is sensitive for the X-ray from 0.2 to 10 keV^7^. Finely binned light curves from 0.3 to 10 keV^44^ during MJD 53485 to 57575 are downloaded from the website^1^, and the light curve is plotted in panel (b) of Figure 1. Following the recommended threads, the UVOT source is processed in HEASoft 6.30.1 with a source circular region radius of $6.5^{\prime \prime }$, and the background is selected from a source-free circular region with radius equal to $30^{\prime \prime }$ in v, b, u, uvw1, uvm2, and uvw2 bands. These light curves are shown in panels (c) and (d) in Figure 1.

### Ground based optical and near infrared data

Optical and near infrared (NIR) observation data are gathered and shown in panel (e) of Figure 1. The source was observed at Steward Observatory from 2008 October 4 to 2018 July 7, by the optical spectropolarimetric monitoring project, which was performed to support the *Fermi* LAT program^42^. Observations in V and R bands are downloaded from the website^2^. This research has made use of up-to-date SMARTS optical/near-infrared observations^3^. These data were observed from MJD 54640 to 57964 in B, V, R, J and K bands, which have already been calibrated and the reduction and analysis are described in^2^.

To conduct the data reduction at bands from ultra-violet (UV) to NIR, we consider the Galactic extinction correction and flux convertion. The galactic extinction is corrected using 0.289 as the $A_V$ value, which is based on the results from^39^ on the NED website^4^. We use the $R_V$=3.1 reddening law^8^ to calculate extinction for photometric magnitudes in NIR, optical and UV bands. Magnitudes are converted into flux densities using the fluxes of zero magnitude given by^1^. The UV and optical spectral index $\alpha _{UV}$ and $\alpha _{O}$ were determined using the power-law model $f_\nu \propto \nu ^{-\alpha }$, where $f_\nu$ represents the flux density and $\alpha$ denotes the spectral index corresponding to the frequency. Here, we employed a linear fit of the form $\log f_\nu = -\alpha \log \nu + C$, with *C* being a constant value. In cases where multiple data points were present at the same wavelength within a single day, we averaged these data, and the standard deviation was calculated and adopted as the error estimate. For the UV spectral index, we consider the cases with all the four bands available on the same day. When the optical spectral index was calculated, we use the criteria that the flux data from at least four out of the B, V, R, J and K bands being available within a single day. The resulting time series of $\alpha _{UV}$ and $\alpha _O$ were plotted in panels (b) and (c) of Fig. 2, respectively.

## Results and explanations

### Variation

From Fig. 1, it is evident that the blazar 3C 454.3 exhibited strong variability during the observation period. From the $\gamma$-ray plot, the object reached its brightest state at MJD 55539.155 with a flux of $1.33 \times 10^{-5} (\pm 1.13 \times 10^{-7})$ $\mathrm{ph\,\, cm^{-2} \, s^{-1}}$ and its faintest state at MJD 59421.155 with a flux of $4.68 (\pm 3.12) \times 10^{-8}$ $\mathrm{ph\,\, cm^{-2} \, s^{-1}}$ in 100-300 MeV band. In the X-ray ($0.3 - 10$ keV) plot, the maximum flux was recorded at MJD 53501.620 with $6.53 (\pm 0.47) \mathrm{counts\,s^{-1}}$, while the minimum flux was observed at MJD 55879.896 with $8.82 (\pm 1.99) \times 10^{-2} \,\mathrm{counts\,s^{-1}}$. The maximum magnitudes observed in various bands were $m_{uvw2} = 16.47 (\pm 0.05)$, $m_{uvm2} = 16.15 (\pm 0.06)$, $m_{uvw1} = 16.30 (\pm 0.07)$, $m_u = 16.41 (\pm 0.06)$, $m_b = 17.14 (\pm 0.18)$, $m_B = 17.34 (\pm 0.12)$, $m_v = 16.51 (\pm 0.09)$, $m_V = 16.76 (\pm 0.10)$, $m_R = 16.62 (\pm 0.02)$, $m_J = 15.09 (\pm 0.02)$, and $m_K = 14.62 (\pm 0.05)$, and the minimum magnitudes were $m_{uvw2} = 13.96 (\pm 0.03)$, $m_{uvm2} = 13.85 (\pm 0.04)$, $m_{uvw1} = 13.49 (\pm 0.04)$, $m_u = 13.27 (\pm 0.03)$, $m_b = 14.04 (\pm 0.03)$, $m_B = 13.96 (\pm 0.002)$, $m_v = 13.45 (\pm 0.03)$, $m_V = 13.44 (\pm 0.03)$, $m_R = 12.95 (\pm 0.02)$, $m_J = 11.01 (\pm 0.004)$, and $m_K = 9.18 (\pm 0.04)$, respectively.

To study the flaring activity at short time scales, we identified six major flares peaking at MJD 55089 (P1), MJD 55176 (P2), MJD 55530 (P3), MJD 56559 (P4), MJD 56829 (P5), and MJD 57558 (P6) from the $\gamma$-ray light curve, labeled with cyan shading in panel (a). These flares were also recorded with complete starting and ending phase in the X-ray, UV, optical, and NIR bands. Since the *R* band light curve has the relative better sampling, we use the variation in *R* band to represent the object’s variations. The time duration, maximun brightness, minimum brightness, the varied magnitude and the number of sub-peaks are summarized in Table 1. The largest varied magnitude $\Delta R = -2.78$ mag over 14 days occurred in the P6 period. After that, the object seems to have entered a prolonged quiescent state from MJD 58000 to MJD 59841, based on the $\gamma$-ray data.

**Table 1:** R band variations.

Period	Duration	Max Brightness	Min Brightness	$\Delta {\textrm{R}}$	Number of
	MJD(day)	(mag)	(mag)	(mag)	sub-peaks
P1	55040.293 - 55099.363	14.04	15.67	1.63	2
P2	55139.994 - 55213.079	13.83	15.29	1.46	1
P3	55478.094 - 55600.102	13.08	15.56	2.48	2
P4	56518.263 - 56578.280	13.38	15.26	1.88	1
P5	56808.422 - 56864.487	12.95	14.66	1.71	1
P6	57547.376 - 57595.352	13.05	15.83	2.78	1

### Time lags

**Fig. 3:**
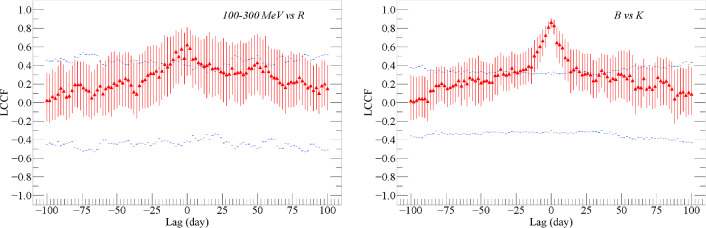
The left panel shows the LCCF results between *Fermi* LAT $100-300 MeV$ and R band fluxes, and the right one shows the LCCF results between *B* and *K* band fluxes. The blue dashed lines indicate the $3\sigma$ confidence levels.

Time lag analysis is important to study the emission mechanism of blazars.^51^ proposed the local cross-correlation function (LCCF), which limits the values of correlation in [-1,1]. We utilize LCCF to get time lags between different wavelengths. The lag range is [-100, 100] days. Considering the best sampling cadence for the optical data is approximately 1 day except the observation gap, we chose a lag bin of 2 days. LCCFs of the *Fermi* LAT $100-300$ MeV versus the optical *R* band fluxes and optical *B* versus *K* band fluxes are shown in the left and right panels of Figure 3, respectively. In order to estimate the significance levels, a Monte Carlo (MC) simulation was used. We simulated ten thousand artificial light curves for B band and $100-300$ MeV fluxes following the method described in^45^. The time bin is 3 days, and the slopes of the power spectral density (PSD) are computed using the Lomb-Scargle periodogram^32^. The two slopes are 0.78 and 1.37 for *B* band and $100-300 MeV$ light curves, respectively. We then extract a subset time series with the same sampling as the observed ones. After that, LCCF between the simulated $\gamma$-ray light curves and the observed *R* (or between simulated *B* and the observed *K*) band light curve are calculated. The $3 \sigma$ confidence level curves are derived by the simulation, corresponding a chance probability of 99.73%. The standard deviation of the correlation for each lag and the standard deviation of time lag are based on the flux randomization (FR) and the random subset selection (RSS) Monte Carlo method proposed by^31^. The peak time delay $\tau _p$ and the centroid time delay $\tau _c$ are considered using the same method in^20^. The errors of time lag correspond to the $1\sigma$ range from the distribution. Here, $\tau {_c}$ is defined as $\tau {_c} = \Sigma {_i} \tau {_i} C {_i} / \Sigma {_i} C{_i}$, and $C{_i}$ are the correlation coefficients greater than 0.8 LCCF($\tau _p$). $\tau _p$ and $\tau _c$ are calculated for ten thousand times to obtain their distributions, and the 1$\sigma$ standard deviations are taken as their errors. In both panels of Figure 3, the peaks of LCCF are beyond the $3 \sigma$ significance level. Time lags $\tau _p = 0.0^{+6.0}_{-8.0}$ and $\tau _c = 0.0^{+5.1}_{-4.4}$ are derived between *Fermi* LAT $100-300$ MeV and *R* band fluxes, $\tau _p = 0.0^{+2.0}_{-2.0}$ and $\tau _c = -0.2^{+2.0}_{-2.2}$ between B and J band fluxes are obtained. Considering the lag bin is 2 days, we note that no significant time lag is found. Meanwhile, because the correlation at zero time lag is over $3 \sigma$ confidence level, we conclude that variations of these bands are co-spatial. This supports the one-zone leptonic models, and is in consistent with the results in^4^.

### Variability timescale

The variability timescale is crucial to understand the underlying emission region size of variability. In order to study its characteristic variability timescale, both the first order structure function (SF,^41^) and the auto correlation function (ACF) are used.

In the case where the time series is given by $(t_j,f_j)$ with arbitrary $t_j$ ($j = 1, 2, ..., n$), the SF can be expressed as^29^:

1 $$\begin{aligned} D_f^1(\tau ,\Delta t) = {1 \over N(\tau ,\Delta t)} \sum _{\tau -\Delta t/2< t_j - t_i < \tau + \Delta t/2}[f_j-f_i]^2, \end{aligned}$$

where $\tau$ is the time lag and $N(\tau ,\Delta t)$ is the number of pairs $[(t_i,f_i ); (t_j,f_j )]$ that satisfy $\tau -\Delta t/2< t_j - t_i < \tau + \Delta t/2$. The squared uncertainty of SF for each $\tau$ was estimated by^41^:

2 $$\begin{aligned} \sigma ^2(\tau ) \approx {{8\sigma ^2_{\delta f} \over {N(\tau )}}} D_f^1(\tau ), \end{aligned}$$

where $\sigma ^2_{\delta f}$ is the measured noise variance. The ACFs are derived using the LCCF method when the two input time series are the same. The timescales can be determined based on the minima of the ACF curve^36^. The timescales can also be determined from the plateau visible in the structure function plot (e.g.^17,29,36^).

Due to the significant correlation and the absence of time lags, the timescale in the *R* band is taken as the representative of the object’s variation timescale. Considering the sampling interval for the *R* band data is approximately 1 day except the observation gap, and considering the duration of variability described in Section "Variation", we calculated the ACFs and SFs using a $\tau$ sample ranging from 2 to 100 days, with a time bin width of 2 days.

**Fig. 4:**
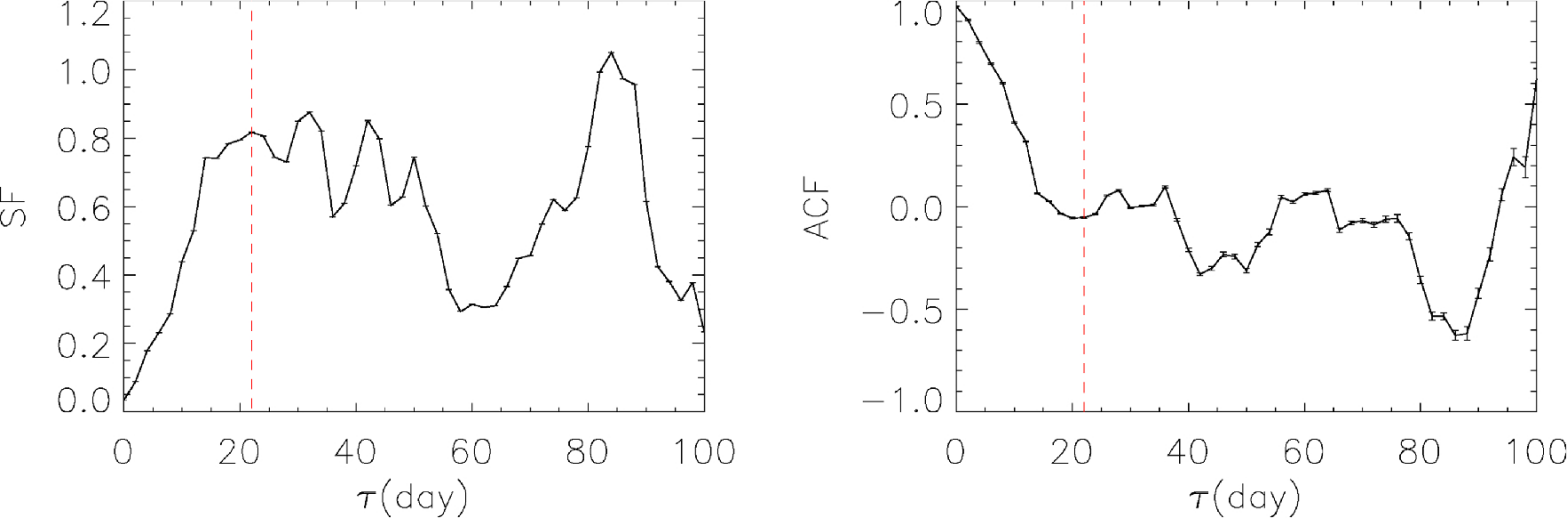
SF and ACF results calculated using *R* band data for 3C 454.3. The dashed line in the left panel marks the SF plateau at a timescale of 22 days, while the dashed line in the right panel indicates the ACF minimum at the same timescale of 22 days.

The results of the SFs and ACFs are shown in Figure 4. A plateau at 22 days is observed in the SF plot, and a local minimum at 22 days is evident in the ACF plot. These features indicate a variability timescale of 22 days.

### Color variation

**Fig. 5:**
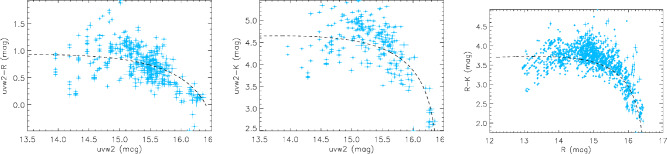
Color variation of 3C 454.3. The cyan diamonds are observation data points, and the dashed lines are the predicted curves using the model stated in Section "The log-parabolic model".

To investigate the color index behaviors, quasi-simultaneous data pairs within the same day between different bands are selected. Considering the frequencies span a narrow range from IR to UV, we selected the uvw2, *R*, and *K* bands as representatives of the UV, optical, and IR bands, respectively. Figure 5 shows the variation in color indices in these bands. The color indices exhibit significant variation, showing an obvious RWB trend when the object is in a lower state, and a saturation phenomenon becomes apparent when the target brightens. This result agrees with many former works^14,33,54,55^. An explanation to the two-stage trend is the existence of the thermal component from the accretion disk, and the saturation stage implies the dominace of jet radiation^14,18,33^.^14^ found it difficult to reproduce color variability by only considering the contamination of the disk radiation for 3C 454.3. We simulated the variation by an analytical model which will be stated in Section "The log-parabolic model".

### Correlations between synchrotron and inverse-compton fluxes

**Fig. 6:**
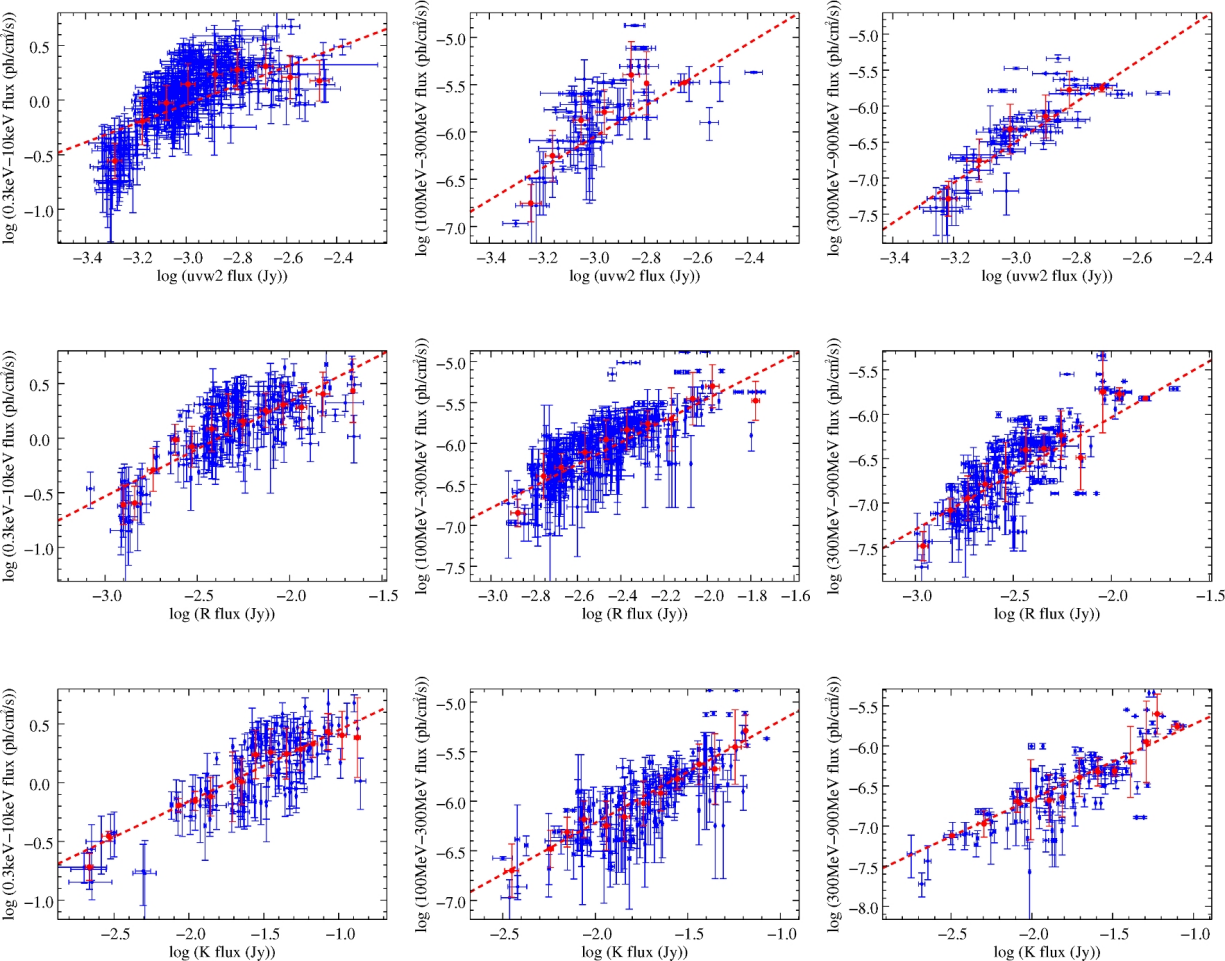
Linear fit of logarithms of synchrotron and inverse-Compton flux. The uvw2, R, and K bands are selected as representatives of the UV, optical, and IR bands, respectively. Each blue circle denote the data pair taken on the same day. The red filled circles denotes the binned data, and the red dashed line indicates the result of linear fitting in each panel.

According to the SED of 3C 454.3^4,15^, we attribute the radiation at *X*-ray and $\gamma$-ray to the IC component, while the UV, optical and IR radiation is of the synchrotron component. As an FSRQ, 3C 454.3 is reasonably believed to have radiation from the accretion disk, the broad line region and the dust torus. Thus, the EC component at the $\gamma$-ray band have rich sources of seed photons. Therefore, we will discuss both the SSC and EC radiation components for this target. Logarithms of the fluxes between the IC and synchrtron component on the same day are plotted in Figure 6. The Swift-XRT $0.3-10$ keV data were binned daily, using the standard deviation as the flux uncertainty. The linear fit is performed with the function

3 $$\begin{aligned} {\log F_{IC} = k * \log F_{syn} + C} \end{aligned}$$

where *C* stands for a constant, *k* is the slope of the linear fit, and $F_{syn}$ and $F_{IC}$ represent the fluxes of the synchrotron and IC components, respectively.

The error weighting is essential when dealing with the correlation of a large number of data points. However, when the data errors were at different levels, the fitting result would be biased in some cases. To address this issue, we have performed binning operations on the data. Starting from the minimum value of $\log F$ in *x*-axis for each diagram, we set a bin size of 0.1. When there are more than three data points in one bin, we calculate its standard deviation and mean value. For bins containing fewer than three points, the corresponding values are not used. The binned data are denoted by the red points in Figure 6. Furthermore, the initial input slope is obtained from the linear fit of unweighted data. Then, the weights can be derived by $w_i = 1/((k\sigma _{x_i})^2+\sigma ^2_{y_i})$. By weighted linear fitting, we obtain new slopes and weights. Finally, we perform another weighted linear fitting algorithm to obtain the output parameters. In Figure 6, the red dash lines represent the linear fitting for the binned data. Pearson correlation coefficients (r-value) between logarithms of synchrotron flux and logarithms of IC flux are calculated, and results are presented in Table 2. For each fit, results are presented in two rows, where the upper one is the slope of the fit, and the lower one is the r-value. The r-values fall within the range from 0.80 to 0.99, indicating that there exist strong correlations in all cases. The slopes range from 0.61 to 3.34 in Table 2. We also notice that the slope roughly decreases as the observing frequency of the synchrotron component decreases.

In the theoretical frame,^9^ presented the dependence of the synchrotron, SSC and EC components on the total number of emitting electrons ($N_e$), magnetic field (*B*), and Doppler factor ($\delta$), which can be writen as:

4 $$\begin{aligned} \begin{aligned} F_{syn} \sim N_eB^{1+\alpha _{syn}}\delta ^{3+\alpha _{syn}},\\ F_{SSC} \sim N_e^2B^{1+\alpha _{syn}}\delta ^{3+\alpha _{SSC}},\\ F_{EC} \sim N_e\delta ^{4+2\alpha _{EC}}U'_{ext}, \end{aligned} \end{aligned}$$

where $\alpha _{syn}$ is the spectral index of synchrotron component, $\alpha _{SSC}$ ($\alpha _{EC}$) is that of SSC(EC) component, and $U'_{ext}$ is the energy density of external photons in the jet comoving frame. If the variation is dominated by a change in *B*, $F_{EC}$ will not vary while $F_{syn} \propto B^{1+\alpha _{syn}}$, resulting in that the $\gamma$-ray and the optical variation will not be correlated, and the slope will be zero. If the variation is due to the change in $N_e$, $F_{EC} \propto F_{syn}$ and $F_{SSC} \propto F^2_{syn}$, and the slope will be 1 and 2, respectively. If a variation is due to the change in $\delta$, $F_{EC} \propto F_{syn}^{(4+2\alpha _{EC})/(3+\alpha _{syn})}$ and $F_{SSC} \propto F_{syn}^{(3+\alpha _{SSC})/(3+\alpha _{syn})}$, and the slope will be $(4+2\alpha _{EC})/(3+\alpha _{syn})$ and $(3+\alpha _{SSC})/(3+\alpha _{syn})$, respectively. By this slope, we can investigate both the emission mechanism at high energy and the variation mechanism at all energy bands.

From Table 2, one can conclude that the observed flux variations are not primarily driven by changes in *B* or $N_e$, since the measured slopes show the dependence on the observing frequency.

In the $\delta$ modulated scenario, the theoretical predicted slope $(4+2\alpha _{EC})/(3+\alpha _{syn})$ and $(3+\alpha _{SSC})/(3+\alpha _{syn})$ can be derived if the spectral index are given. As depicted in Figure 2, the spectral index for $\gamma$-ray varies between 0.04 and 2.15, averaging 1.18 with a standard deviation of 0.35. The UV spectral index spans from 0.52 to 1.82, featuring an average of 0.99 and a standard deviation of 0.19. The optical spectral index ranges from -0.09 to 2.31, with an average value of 1.37 and a standard deviation of 0.37. Thus, by inserting the spectral indices and considering all possible cases, the range of slope for the SSC process versus optical (or UV) can be derived as [0.57, 1.77] (or [0.63, 1.46]). For the EC process versus optical case, the range of slope values is [0.77, 2.85]. And the slope values is [0.85, 2.36] for the EC process versus UV case.

We notice that the slope for both the SSC and EC versus UV case could not explain the observed slope for the 300-900 MeV versus UV case. This may be due that there may be other emission components like corona or disk at UV bands. We also observe that the predicted slopes of EC versus optical matches better than that of SSC versus optical. Thus, the case that EC process is dominant at $\gamma$-ray is favored for 3C 454.3, which is also consistent with the broadband SED fitting (e.g.^15^). From above analysis, we also conclude that the variation mechanism of the target is mainly due to the Doppler factor. Other variation mechanism like the particle evolutions in the shock-in-jet model could also be possible, but an analytical slope is still unclear.

**Table 2:** Linear fit results for logarithms of the synchrotron and IC flux.

*band*	*uvw*2	*uvm*2	*uvw*1	*u*	*b*	*B*	*v*	*V*	*R*	*J*	*K*
$0.3 - 10 keV$	0.87	0.93	0.86	0.77	0.69	1.01	0.78	0.87	0.87	0.89	0.61
	0.80	0.81	0.88	0.86	0.84	0.91	0.86	0.92	0.92	0.97	0.99
$100 - 300 MeV$	1.65	1.86	1.53	1.43	1.51	1.41	1.57	0.96	1.34	1.14	1.03
	0.94	0.88	0.92	0.96	0.98	0.98	0.96	0.97	0.95	0.97	0.99
$300 - 900 MeV$	2.78	3.34	2.67	2.28	1.99	1.73	1.98	1.16	1.27	1.24	0.93
	0.97	0.99	0.98	0.98	0.98	0.98	0.97	0.92	0.98	0.99	0.99

### The log-parabolic model

Based on the analysis in Section "Correlations between synchrotron and inverse-compton fluxes", we use the correlations to conclude that the flux variation is possibly dominated by the Doppler factor $\delta$. To understand the variation in a quantitative manner, we use a log-parabolic function to describe its synchrotron emission phenomenologically^27^, i.e.

5 $$\begin{aligned} {\log (\nu ' F_{\nu '}) = \log ({\nu '_{P}F_{\nu '_{P}}) - b [\log (\frac{\nu '}{\nu '_{P}} )]^2}}, \end{aligned}$$

where $\nu '_{P}$, $F_{\nu '_{P}}$, and *b* are free parameters, in which $\nu '_{P}$ is the peak frequency of the emissivity in rest frame of the emitting source, $F_{\nu '_{P}}$ is the observed flux at $\nu '_{P}$, *b* is the curvature, and $\nu '_{P} F_{\nu '_{P}}$ can be regard as an overall constant amplitude factor.^43^ obtained the Doppler boosted flux of synchrotron emission in the observer frame

6 $$\begin{aligned} { \nu F_{\nu } \over {\nu '_{P}}F_{\nu '_{P}}} = \delta ^{3} \exp \{-b [ \log ({\nu \over \delta \cdot \nu '_P})]^2 \}. \end{aligned}$$

In addition, we assume that there is a relative stable background contamination (BC) to the flux, and it is lower than the observed flux in its faintest state in history data. Then the observed flux can be written as

7 $$\begin{aligned} F_{\nu }^{obs} = {F_{\nu }} + BC_{\nu } \end{aligned}$$

where $BC_{\nu }$ is the BC value at the frequency $\nu$.

Based on the scenario described above, we try to find a solution using the data paris within 1 day.^10^ fitted the SEDs of blazars using a log-parabolic law and derived the peak frequency, $\nu _P$, in the observer’s frame. For 3C 454.3, the curvature *b* and $\nu _P$ were found to be 0.233 and $10^{12.791}$, respectively. Given that $\nu _P = \delta \nu '_P$, and considering that the Doppler factor $\delta$ varies within the range [8.8, 36.14] for 3C 454.3^13^, we set that $\nu '_P$ lie within [$10^{11.0}, 10^{12.5}$]. The parameter *b* is set within the range [0.02, 0.5] to encompass its true value. To ensure that our analysis covers the likely true value of the parameter *b*, it is set within the range [0.02, 0.5]. When considering Equation 7, we set the ratio of $BC_R$ over the observed faintest brightness in R band within [0.02, 1]. According to^23^, FSRQs have a median value of $\delta \approx 11$, and a maximum of $\delta \approx 60$. Thus, we set the Doppler factor $\delta$ to vary within the range [$1.05^{0}$, $1.05^{100}$] to encompass these values. In order to decrease the calculation time, we binned data for every 0.1 magnitude from 12.95 to 16.45 in *R* band, and the corresponding quasi-simultaneous data in *V* and *B* bands are binned. Calculations are organized in the following steps:

1. The peak frequency $\nu '_P$ is given in the range from $10^{11.0}$ to $10^{12.5}$, with a power step of 0.02.
2. Curvature *b* is assigned a value between 0.02 and 0.50, with a step of 0.02;
3. $BC_R$ ranges from 2 to 100 percent of the flux minimum of 3C 454.3 in observation, with a step of 2 percent.
4. The Doppler factor $\delta$ varies in the range [$1.05^{0}$, $1.05^{100}$] with a power step of 1.
5. Given a set of $BC_R$, $\nu '_P$, *b* and $\delta _{min}$, the amplitude factor $\nu '_{P} F_{\nu '_{P}}$ is calculated according to the minimum flux in observations.
6. Then the Doppler-boosted flux $F_{\nu }$ in *B*, *V* and *R* bands can be derived for other $\delta$ values.
7. By anchoring the predicted $F_{R}$ with $F_{R}^{obs}$, the Doppler factor for $F_{R}^{obs}$ can be obtained. Then, we predict $F_B$ and $F_V$ to derive $BC_B$ and $BC_V$ for each data pair.
8. Finally, the variances of $BC_B$ and $BC_V$ ($S^2=\Sigma _{i=1}^N (BC_i-<BC>)^2/(N-1)$) are calculated. When the sum of variances gets its minimum, we obtain the best fit result.
9. This process is repeated for ten thousand times to get the error estimates by using the FR method^31^.

Parameters of the best fit result are listed in Table 3. The $BC_R$ is $1.03 \pm 0.46$ mJy, which means that the magnitude of the source would be $16.42^{+0.64}_{-0.40}$ when $F_{\nu }$ equals to 0. We are also able to derive the average value of *BC* in other synchrotron radiation band using simultaneous data between *R* and other bands. The $BC_{\nu }$ and its $1\sigma$ uncertainty are listed in Table 4. The magnitudes $Mag_\nu$ are also calculated corresponding to the average $BC_{\nu }$ fluxes without considering $BC_{\nu }$ error.

**Fig. 7:**
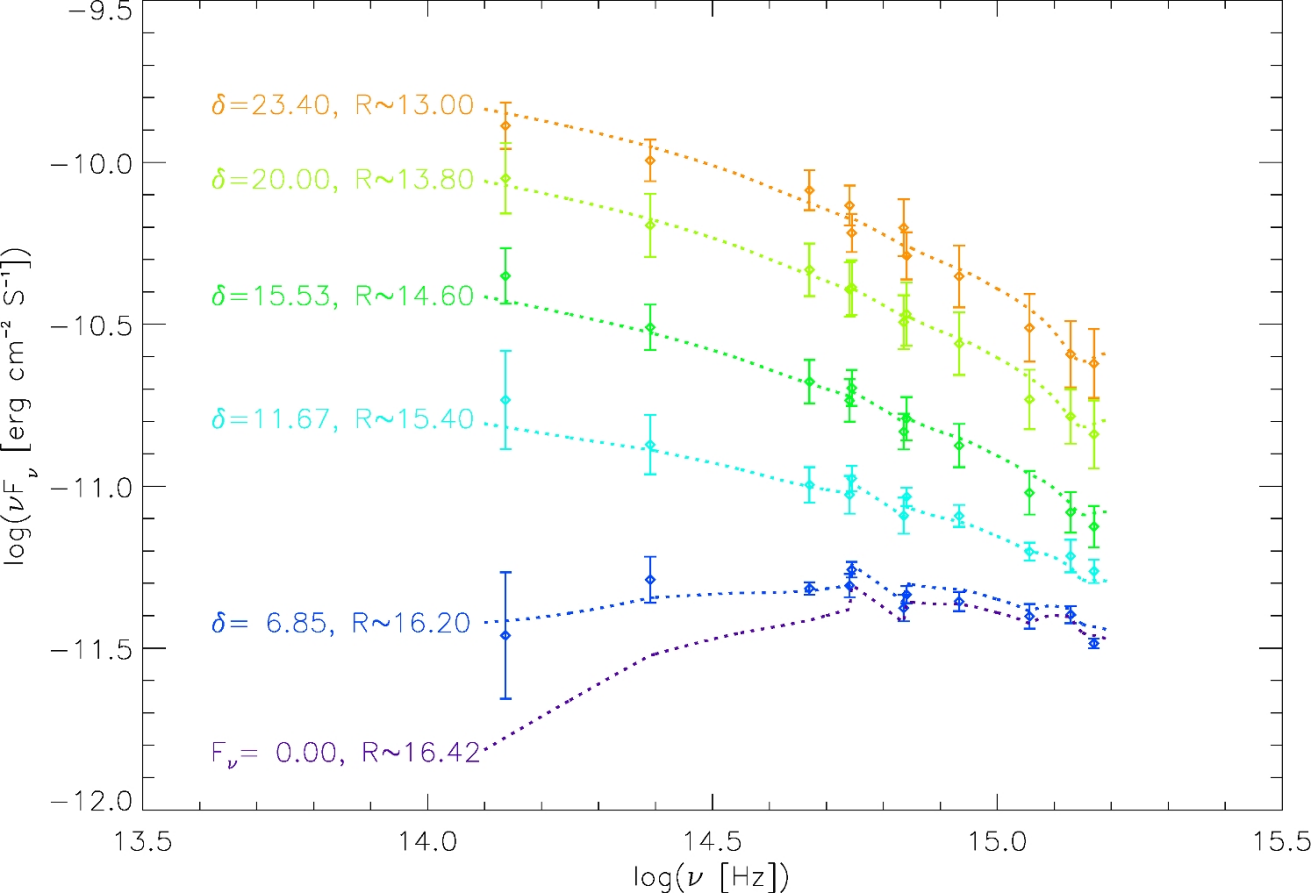
Fit to the SED of 3C 454.3 using our model during different states. Doppler factor were selected when the R magnitude was between 13 and 16.2, with intervals of 0.8 magnitudes and a precision within 0.05 magnitudes.

Using these parameters, we predict the fluxes in the observed bands as Doppler factor changes, as indicated by the dash lines in Figure 7. Diamonds in Figure 7 show simultaneous data points where the *R* magnitude ranging from 13.0 to 16.2 with a step of 0.8. It is clear that the predicted SED fit the observation data well. The obtained *BC* components are indicated by the bottom dash lines, which could represent the contribution of non-jet components. The predicted color indices are plotted in Figure 5 with black dashed lines.

**Table 3:** Best fit parameters for synchrotron emissions.

parameters	$\log (\nu '_P (Hz))$	*b*	$BC_R(mJy)$
*Values*	$12.04^{+0.02}_{-0.02}$	$0.36^{+0.02}_{-0.06}$	$1.03^{+0.46}_{-0.46}$

**Table 4:** $BC_{\nu }$ in synchrotron bands.

*Band*	*uvw*2	*uvm*2	*uvw*1	*u*	*b*	*B*	*v*	*V*	*J*	*K*
$BC_{\nu }$(mJy)	0.51	0.67	0.58	0.77	0.91	0.79	1.17	0.99	1.32	1.26
*Error*(mJy)	0.03	0.05	0.05	0.06	0.05	0.03	0.11	0.03	0.24	0.84
$Mag_\nu$	16.29	16.04	16.13	16.18	17.01	17.20	16.51	16.72	15.12	13.92

Given the observed variability timescale of 22 days as derived in Section "Variability timescale", which corresponds the light travel time of 11.8 days (redshift corrected) across the radiating region, it is plausible that the radiation originates from a jet, producing the beaming effect. Additionally, the observation of zero time lags between the light curves in different bands suggests that the variability is chromatically neutral or “white,” supporting the possibility of Doppler factor $\delta$ variations within this source.

Many former works estimated $\delta$ in 3C 454.3, and their results showed that $\delta$ varies at different epochs (e.g.^13^ and^23^), which support the $\delta$ modulated variation scenario.^21^ compared SEDs of the source for 4 periods, and found that the Doppler factor is larger when a larger variability amplitude happens.^13^ censused $\delta$ value of 3C 454.3 in literature ranging from 8.8 to 36.14. Our result is in accordance with these historical results.^24^ interpreted the two flares in 2013 and 2014 of 3C 454.3 with a shock propagating down the jet. The flare in 2013 originated due to changes in the viewing angle, and the shock crossing was responsible for the other one.^14^ found it difficult to produce the observed color variability by only considering the contamination of disk radiation, while Doppler factor variation was not regarded as a main reason for the variability.^37^ modelled the SEDs of 3C 454.3 with a one-zone leptonic model considering IC of synchrotron, disc, and BLR photons, and found that the Doppler factor substantially increased during the flares.^15^ found the $\gamma$-ray flaring activity of 3C 454.3 is mainly caused by the Doppler factor. Our results are in agreement with the cited works.

In^28^, they assumed that a relativistic jet aimed to the line of sight at a small angle $\theta$. The radiation at the optical band was of synchrotron process. The Doppler factor is $\delta = {1 / \Gamma (1-\beta cos\theta })$, where $\Gamma = 1/\sqrt{1-\beta ^2}$ is the Lorentz factor of the bulk motion of the emitting plasma. They present that

8 $$\begin{aligned} dm/dt_{obs} = (3+\alpha )\beta \Gamma \delta ^2 \sin \theta (d\theta / dt_{jet}), \end{aligned}$$

where $dm/dt_{obs}$ denotes the observed variation rate of magnitude, $\alpha$ is the spectral index. For 3C 454.3,^23^ derived that $\theta$ ranges from 0.01 to 2.15 with an average value of 1.79, and $\Gamma$ ranges from 13.32 to 42.94 with an average value of 17.13. Considering that the max amplitude in brightness variation was about 2.78 mag (P6 in table 1) with the timescale of 7.5 days(redshift corrected), we derive that the $dm/dt \approx 0.015$ mag per hour. Hence, using the average value of $\Gamma$ and the minimum value of $\theta$, we estimate that $d\theta / dt_{jet} \approx 2.5$ arcsec per hour for the brightest state ($\delta =23.4$) of the target.

However, the scenario described above is not the only possible process to explain the observations, and the scenario is not sufficient to interpret the scatter for a bright state in the color index diagrams.^4^ explained the observations by stochastic injection of energy in jet plasma from the central engine and dissipation at different distances from the base of the jet. The variation could also be due to changes in $N_e$, $\delta$ or a combination of one or both with changes in B^9^. Other intrinsic variation mechanisms, with proper parameter tuning, may also be capable of explaining its spectral variation^52^. Nevertheless, the Doppler factor variation is indeed an important factor for different amplitude of outbursts on the long-term timescale.

## Conclusion

The long-term multi-wavelength light curves of 3C 454.3 are gathered and reduced. We analysed the time lags and correlations between different bands to distinguish whether the radiation from the same region or the same mechanism. Based on the LCCF analysis, no significant time lag is found between $\gamma$-ray $100-300$ MeV and optical *R* band, neither between *B* and *K* bands. It is credible that emissions in these bands are from the same radiation region.

The color index variation and the correlations between the synchrotron versus IC components are studied. Color indices show a RWB trend when the source is faint, and a saturation effect occurs when the source is at a high flux level. The flux of the IC component is correlated with that of the synchrotron component in the log-log plot, and the slopes range from 0.61 to 3.34. We propose a model that is composed of a stable $BC_{\nu }$ and a Doppler boosted log-parabolic synchrotron emission. By using a fitting algorithm, the $BC_{\nu }$ at UV, optical, and IR bands are derived. Using this model, we find that the long-term varied SED can be fitted well by considering variations of the Doppler factor. If the Doppler boosted scenario is true in 3C 454.3, one can utilize parameters derived above to predict the Doppler factor using the observed brightness at arbitrary optical band, especially when the source is in a bright state. Our method can also be used in other sources whose brightness is dominated by Doppler factor variation. However, there is a caveat that our method is model dependent. If the BC is also variable, we should refrain from apply it.

## Data Availability

All the data in this work were obtained from online sources, with their origins appropriately referred within the text.

## Footnotes

1 http://www.swift.psu.edu/monitoring/

2 http://james.as.arizona.edu/~psmith/Fermi/

3 http://www.astro.yale.edu/smarts/glast/home.php

4 http://ned.ipac.caltech.edu/
